# Pancreatic schwannoma: a rare differential diagnosis for a pancreatic mass

**DOI:** 10.1259/bjrcr.20230029

**Published:** 2023-08-02

**Authors:** Dana AlNuaimi, Shareefa Abdulghaffar, Giuseppe Iuppa, Ahmad AlDuaij, Noor Badrawi, Numan Cem Balci

**Affiliations:** 1 Department of Health, Abu Dhabi, United Arab Emirates; 2 Dubai Health Authority, Dubai, United Arab Emirates; 3 Cleveland Clinic Abu Dhabi, AlMaryah Island, Abu Dhabi, United Arab Emirates; 4 Cleveland Clinic Lerner College of Medicine, Ohio, United States

## Abstract

Pancreatic schwannomas are rare benign tumors with low malignant potential and are often difficult to diagnose due to their non-specific presenting symptoms and overlapping radiological imaging characteristics. Cross-sectional imaging plays an important role in the initial diagnosis and in delineating the extent of the lesion. However, biopsy and histopathological examination remains the gold-standard for a definite diagnosis. The management of pancreatic schwannomas includes surgical resection often yielding excellent clinical outcomes with low recurrence rates. We present a case of a 33-year-old female patient with a history of a recurrent vague upper abdominal pain where CT of the upper abdomen showed a hypodense pancreatic mass. Robotic subtotal pancreatectomy was done with histopathology showing spindled Schwann cells indicative of a pancreatic schwannoma.

## Introduction

Intra-abdominal schwannomas are a rare type of mesenchymal peripheral nerve tumors that arise from Schwann cells.^1^ Pancreatic schwannomas are even more uncommon accounting for less than 1% of all schwannomas and usually develop from Schwann cells lining the branches of the vagal nerve that courses through the pancreatic tissues.^1,2^ Patients usually present with variable symptoms including abdominal or back pain, nausea, vomiting, weight loss and jaundice and non-specific radiological features making pre-operative diagnosis difficult.^3,4^ Pancreatic schwannomas are a rare differential diagnosis of both solid and cystic masses arising in the pancreas.^5^

## Case report

A 33-year-old female patient presented at the outpatient gastroenterology clinic complaining of vague upper abdominal pain for the past 2 years. The pain is intermittent and colicky in nature and often radiates to the flanks. No correlation with food intake was noted nor specific relieving or aggravating factors were identified. No significant past medical or surgical history was given. No history of smoking or alcohol intake. No family history of pancreatic masses.

**Figure 1.**
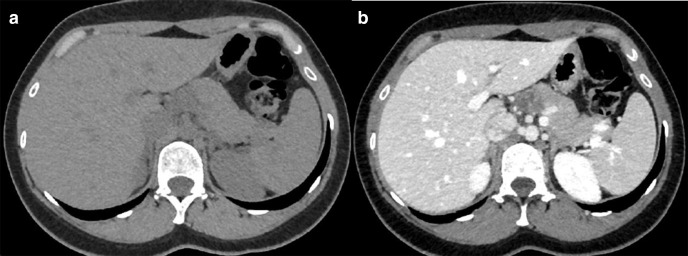
CT scan of the upper abdomen. (a). Non-enhanced axial section showing a hypodense 23 x 21 mm lesion in the pancreatic neck in relation to celiac trunk. (b). Contrast-enhanced axial section showing mottled enhancement of the pancreatic mass.

On examination, the patient’s vital signs were within normal limits. Chest was clear and abdomen was soft with no palpable masses. The patient had undergone an abdominal ultrasound and a contrast-enhanced CT scan of the abdomen and pelvis in another healthcare facility revealing a 2.3 × 2.1 cm mass at the neck of the pancreas. The lesion had multiple septations and appeared hypodense in comparison to the rest of the pancreatic tissues giving a mottled appearance after contrast administration. No dilatation of the pancreatic duct was seen (Figure 1).

**Figure 2.**
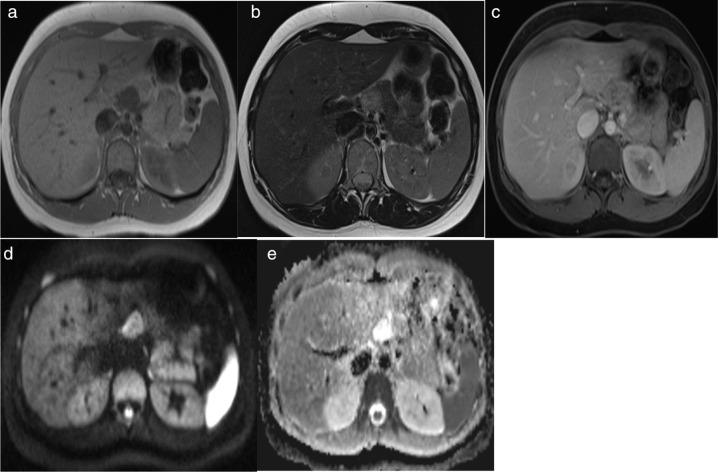
MRI of the upper abdomen at the level of the pancreas. (a) Non-enhanced axial *T*_1_WI showing a hypointense 23 x 21 mm lesion in the pancreatic neck. (b) Axial *T*_2_WI showing hyperintense appearance of the lesion (c) Gadolinium-enhanced axial *T*_1_WI showing mildly mottled enhancement of the lesion. (d, e) DWI and ADC showing no diffusion restriction. ADC, apparent diffusion coefficient; DWI, diffusion-weighted imaging.

MRI of the abdomen was concurrently done showing the mass at the neck of the pancreas which appeared hypointense on *T*_1_ weighted images and hyperintense on *T*_2_ weighted images in comparison to the pancreatic tissues with mild mottled enhancement upon gadolinium contrast administration and no evident restriction on diffusion weight images and apparent diffusion coefficient (Figure 2).

**Figure 3.**
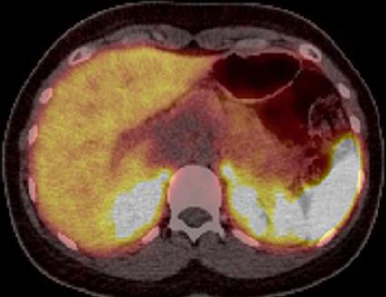
Gallium 67-DOTA PET-CT revealing no significant tracer uptake of the pancreatic lesion. PET, positron emission tomography.

The differential diagnosis included adenocarcinoma, serous and mucinous cystic neoplasms as well as solid pseudopapillary epithelial neoplasms (SPEN) considering the patients age and gender. A 68 Gallium PET-CT dotatate scan to identify neuroendocrine tumors was done and yielded negative results (Figure 3).

Complete blood count and a comprehensive metabolic laboratory panel was done. Pancreatic enzymes were not elevated, Ca-19–9 and chromogranin A for neuroendocrine tumors yielded negative results.

The patient was advised to undergo endoscopic ultrasonography and biopsy. However, the patient opted for surgical resection of the mass and refused biopsy. Surgical complications such as post-operative infections, intraoperative bleeding, vascular injury and pancreatic insufficiency were explained thoroughly to the patient.

Distal robotic subtotal pancreatectomy was done. An exophytic rounded and smooth-surfaced firm mass was noted at the neck of the pancreas and was adherent to the splenic artery and celiac trunk. The mass was carefully dissected and mobilized preserving the splenic vasculature. A peripancreatic reactive lymph node was also resected.

**Figure 4.**
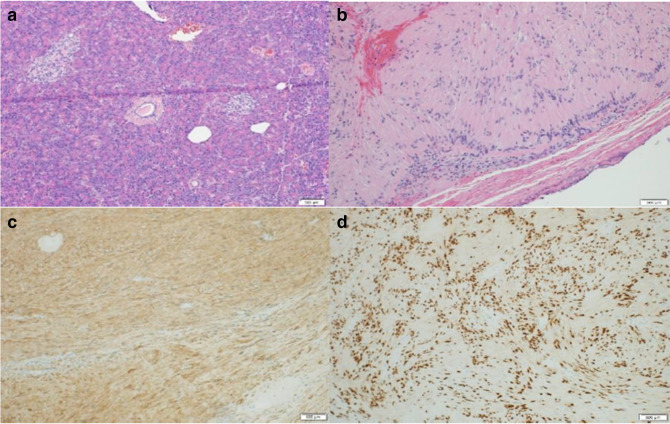
Histologic sections of the pancreatic mass. (a) Spindled Schwann cells with Antoni A and Antoni B areas. (b) Hematoxylin and Eosin staining showing focal degenerative changes with nuclear pleomorphism and focal cystic degeneration. No significant increase in mitotic figures seen. (c, d) Immunohistochemical stains show tumor cells expressing S100 and SOX10. Original magnification x40 (a), x100 (b, c and d).

Pathology showed a well circumscribed grayish-white mass approximately 2.3 × 2.1 cm in diameter composed of spindled Schwan cells with Antoni A (cellular, fascicular) and Antoni B (myxoid, vacuolated) areas. Focal degenerative changes including cystic changes were seen. No significant increase in mitotic figures. Immunostains showed tumor cells expressing S100 (diffuse) and SOX10. The spindled cells were negative in SMA, CD117 and DOG1 stains. Proliferation rate index by ki67 stain was variable of 0–8% nuclear staining focally (Figure 4).

The patient had an unremarkable recovery with no complications and was referred for follow-up at the gastroenterology and endocrinology outpatient clinics.

## Discussion

Schwannomas were first described in 1910 as spindled-cell tumors that originate from the myelin producing Schwann cells lining the peripheral nerve sheaths of autonomic sympathetic or parasympathetic fibers.^1,3,5^ They are usually seen in the extremities, head and neck, mediastinum and in the retroperitoneum with less than 100 cases of pancreatic schwannomas reported in the literature up to the current date.^3,6^

Pancreatic schwannomas are typically benign encapsulated tumors with only a few reported cases of malignant transformation. They are commonly seen in the head of the pancreas in almost 40% of the cases and in the body of the pancreas in 20% of cases.^1–3^

They are more common in females than males and are usually seen in the third to fifth decade of life.^2^ Patients are asymptomatic in 30% of the cases. Approximately, 70% of patients present with non-specific symptoms including upper abdominal or back pain, nausea, vomiting, weight loss and jaundice.^2,7^

Pancreatic schwannomas are often discovered incidentally on CT or MRI as solitary pancreatic masses. However, they may rarely present as multiple masses when associated with Von Recklinghausen’s disease.^3,8^ They are often well-circumscribed with solid, cystic or heterogenous appearance on diagnostic imaging depending on their histological pattern (Antoni A or Antoni B).^4–6,8,9^

Schwannomas with predominantly Antoni A (hypercellular) areas are usually seen on CT as inhomogeneous, hypodense solid tumors that enhance post-contrast administration while those with predominantly Antoni B (hypocellular) areas are seen as well-defined homogenous cystic lesions with no significant contrast enhancement.^4,5,8,10^

On MRI, pancreatic schwannomas usually exhibit low signal intensity on *T*_1_ weighted images and high signal intensity on *T*_2_ weighted images.^8^ Larger lesions may undergo degeneration with cystic changes, calcifications, hemorrhage and hyalinization appearing more heterogenous and complex and mimicking other pancreatic tumors.^8^

On diagnostic imaging, the differential diagnosis of cystic pancreatic schwannomas includes mucinous or serous cystadenomas, intraductal mucinous papillary tumors and pancreatic pseudocysts. Solid pancreatic schwannomas may mimic neuroendocrine tumors and pancreatic adenocarcinomas.^2,10^ Nevertheless, for a definitive diagnosis of pancreatic schwannomas a biopsy and histopathological examination showing areas of Antoni A and Antoni B as well as exhibiting a diffuse strong staining for S-100 remains the gold-standard.^5^

The management of pancreatic schwannomas may include simple enucleation for smaller lesions and for those showings benign histological cell types on intraoperative frozen sections. However, for masses showing malignant characteristics a pancreatoduodenectomy or distal pancreatectomy with or without splenectomy is often necessary.^5,10^

## Learning points

- Pancreatic Schwannomas are rare benign lesions that may appear as solid, cystic or heterogenous masses depending on their histology and may mimic other pancreatic tumors. Intraoperative histopathological examination of the frozen sections is crucial in aiding the surgical decision from simple enucleation to radical resection.
- Diagnostic imaging plays an important role in establishing the diagnosis, defining the nature of the lesion and in delineating its extent. Nevertheless, for a definite diagnosis histopathological examination remains the gold-standard.
